# Stereotactic Body Radiation Therapy for Symptomatic Benign Spinal Tumors: A Single-Center Retrospective Experience

**DOI:** 10.1016/j.adro.2026.102127

**Published:** 2026-09-11

**Authors:** Matthew S. Susko, Jonathan Massachi, Lauren Boreta, Jessica Chew, Harish Vasudevan, Jean Nakamura, Steve E. Braunstein

**Affiliations:** Department of Radiation Oncology, University of California, San Francisco, California, United States

## Abstract

**Purpose:**

Benign spinal tumors (BSTs) are relatively uncommon, though can yield significant morbidity as related to location and size. Surgical resection is the primary management modality for symptomatic lesions; however, for patients who are nonsurgical candidates or have progressed post-surgery, radiation therapy can be employed for definitive treatment. This institutional series describes the local control and toxicity outcomes of stereotactic body radiation therapy (SBRT) for BST.

**Methods and Materials:**

Patients treated with SBRT for BST at a single institution from 2010 to 2021 were retrospectively reviewed and analyzed. Patients were included if they underwent treatment with SBRT with or without prior surgical intervention. Evaluation of posttreatment toxicities, symptomatic improvement, imaging response, and local control were performed.

**Results:**

During the study period 31 patients were identified with a total of 36 BST. The distribution of the tumors included 16 in the cervical spine (44%), 13 thoracic (n = 36%), and 7 lumbosacral (n = 20%). Histologies included 14 schwannoma (39%), 12 meningioma (33%), and 8 hemangioma (22%), 1 melanocytic tumor (3%), and 1 neurofibroma (3%). Median radiation prescription dose was 25 Gy (range, 18-40 Gy) in 5 fractions (range, 3-5 fractions). Of the treated lesions, 17 (47%) had prior surgical management and 19 (53%) were treated upfront with SBRT. With median imaging follow-up was 49 months (range, 2-171) and the median clinical follow-up was 83 months (range, 10-171) with 86% of assessable patients having stable to improved symptoms in the treated lesion at 12 months. Two patients demonstrated progressive disease on imaging. There were no incidences of National Cancer Institute Common Terminology Criteria for Adverse Events version 4 grade 3 or greater observed toxicities and the most common toxicities were fatigue 6 (17%), and pain flare 5 (14%), both transient. There was no association between treatment course (prior surgery versus definitive radiation) and symptomatic stability or improvement, imaging response, or toxicity.

**Conclusions:**

SBRT is well tolerated for treatment of symptomatic BST with high rates of symptomatic stability or improvement. This series demonstrated acceptable local control and no high-grade toxicities.

## Introduction

Primary tumors of the spinal column are rare, representing only 4% to 8% of all central nervous system tumors. Benign spinal tumors (BSTs), including meningioma and nerve sheath tumors, account for 60% to 70% of primary spinal tumors and often result in significant morbidity due to neurologic symptoms related to localized mass effect.^1^, ^2^, ^3^ For this reason surgical management for BST has been the preferred treatment option for symptomatic lesions, resulting in durable local control, the potential for immediate symptomatic relief, and modest morbidity.^4^

Stereotactic body radiation therapy (SBRT) has been used to treat these benign lesions noninvasively in a small number of reports as either a definitive treatment, or as an adjunct to surgery, particularly in instances of partial resection or recurrence.^5^, ^6^, ^7^, ^8^ SBRT is known to have excellent dose distribution with delivery of high doses of radiation to the target and relative sparing of adjacent tissues to within thresholds of organ-at-risk tolerance.^9^

The primary dose limiting structures during the treatment of BST are the parenchymal spinal cord and nerve roots. The use of SBRT is particularly advantageous in this setting because of the steep dose gradients which helps ensure ablative doses of radiation to the target, while maintaining safe doses to normal tissues.^10^, ^11^, ^12^, ^13^ This is of particular importance in symptomatic BST as these lesions are typically in close approximation to the spinal cord and nerve roots, making precise dosimetry and treatment delivery of utmost importance in maintaining therapeutic effectiveness and minimizing toxicity risk.

Given the limited published experiences for the use of SBRT for symptomatic BST, particularly in regards to longer-term follow-up,^7^^,^^8^ we performed a retrospective analysis of all patients who were treated for symptomatic BSTs at our center between 2010 and 2021 focusing on the outcomes of symptomatic improvement, radiographic response to treatment, and toxicity.

## Methods

In this institutional review board–approved retrospective analysis, patients treated at a single academic center for BST from 2010 to 2021 were retrospectively analyzed. BSTs included meningioma, hemangioma, schwannoma, neurofibroma, and other entities based on radiologic and/or pathology diagnosis. Hemangiomas were included if there was symptomatic epidural or neuroforaminal extension but not asymptomatic lesions or exclusively interosseous lesions. Patients were allowed to have prior surgical resection but were included only if there was symptomatic residual or recurrent disease. Clinical data were extracted from the electronic health record, including patient demographics, tumor and treatment characteristics, presenting symptom and symptom burden at last follow-up. Treatment of multiple noncontinuous sites were considered different lesions for analysis. Data on patients undergoing radiologic follow-up were assessed for local control and progression after treatment delivery.

SBRT was delivered with immobilization and daily image guidance using either a nonisocentric or isocentric linear accelerator delivery with ablative intent. The maximum number of allowed fractions was 5 with the specific dose and fractionation scheme at the discretion of the treating physician. Dose constraints and planning objectives for SBRT treatments were derived from American Association of Physicists in Medicine Task Group 101 and High Dose per Fraction, Hypofractionated Treatment Effects in Clinic (HyTEC).^14^

Statistical analysis was performed with the use of RStudio (RStudio, Boston, Massachusetts, USA). Descriptive statistics included median, IQR, and ranges were considered for both per patient and per lesion basis. Chi-square tests for proportions and univariate logistic regression were used to assess for differences in outcomes between the radiation only and prior surgery groups based on categorical and continuous variables. Toxicities were assessed retrospectively using the National Cancer Institute Common Terminology Criteria for Adverse Events (version 5.0) criteria. The Kaplan-Meier method was used for calculation of progression-free survival (PFS) of the lesions after treatment with SBRT.

## Results

Between 2010 and 2021, 31 patients were identified with 36 symptomatic BST treated with SBRT. Demographic features and tumor characteristics can be found in Table 1. Median age of patients was 57 years (range, 16-80 years) and median Karnofsky performance status as 90 (range, 70-100). Tumors were distributed across all spinal levels with 16 lesions centered in the cervical spine (44%), 13 in the thoracic spine (36%), and 7 in the lumbosacral spine (20%). The most common presenting symptom in this group was pain (89%), with sensory changes (36%) and weakness (25%) also being noted as presenting symptoms.

**Table 1:** Demographic and tumor characteristics

Characteristic	Value
Age (y), median [IQR]	57 [43-63]
Sex (Biological)	
Male	11 (35%)
Female	20 (65%)
Karnofsky performance status, median [IQR]	80 [70-90]
Presenting symptoms	
Pain	32 (89%)
Paresthesia	13 (36%)
Weakness	9 (25%)
Histology	
Meningioma	12 (33%)
Hemangioma	8 (22%)
Schwannoma	14 (39%)
Other	2 (5%)
Spine level	
Cervical	16 (44%)
Thoracic	13 (36%)
Lumbosacral	7 (19%)
Radiation intent	
Definitive	19 (53%)
Adjuvant	15 (42%)
Recurrent	2 (5%)

Of the 36 lesions, 17 (47%) had previously undergone surgery prior to receipt of radiation therapy. None of the lesions had been treated previously with radiation therapy. The median radiation dose was 25 Gy (range, 18-40 Gy) treated in a median of 5 fractions (range, 3-5 fractions) with a median Biologically Effective Dose (BED_3_) (α/β=3) of 66.7 Gy (range, 46.7-146.7 Gy). Of the target volumes the median planning tumor volume size was 6.0 cm^3^ (IQR, 3.5-19.4 cm^3^). Clinical follow-up was a median of 83 months (range, 10-171 months), with median magnetic resonance imaging follow-up of 49 months (range, 2-171 months).

After treatment, patients were recommended for clinical follow-up and imaging every 3 to 6 months for the first 2 years, then annually thereafter. After completion of SBRT, 21 (58%) patients noted symptomatic improvement at the treated sites, with 12 (33%) being assessed as having stable symptoms, and 3 (9%) of the treated sites having worsening of symptoms over time. The median time-to-symptom improvement was 4 months with a range of 1 to 10 months. There was no significant difference in symptomatic improvement between patients who had prior surgical resection (53%) and patients treated with radiation therapy alone (63%) (*P* = .53). There was no association between radiation therapy dose or anatomic site of the lesion and symptom improvement.

**Figure 1:**
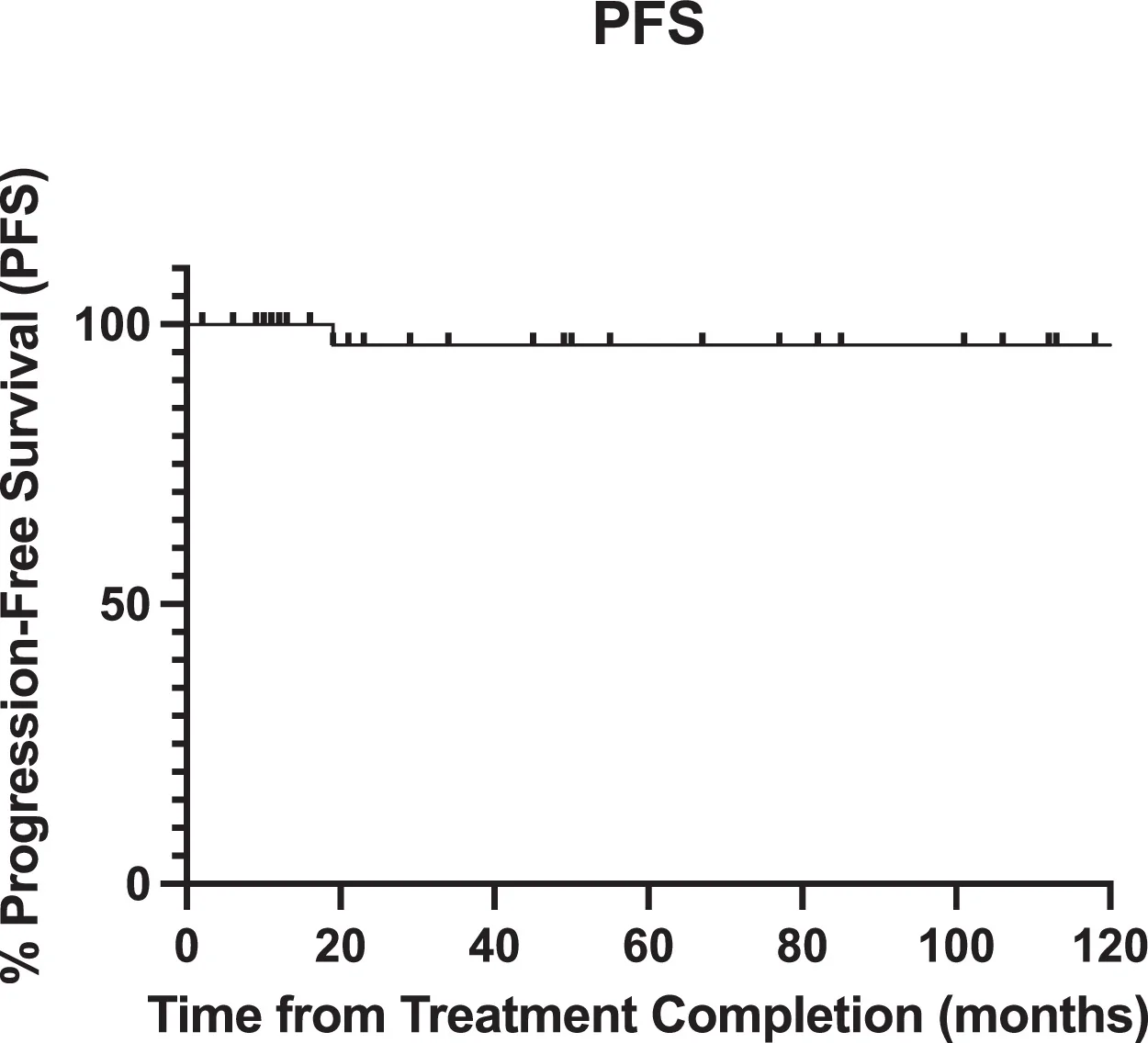
Kaplan-Meier curve of progression-free survival. No events of radiographic disease progression were noted although there were 2 reports of slight tumor enlargement because of treatment effect. Circles represent censoring at the time of the last follow-up.

There were 2 cases of local failure or progressive disease at the treated sites with 2-year PFS of 100%, and 5-year PFS of 97%, with the additional local failure occurring at 135 months. On imaging follow-up 2 lesions demonstrated posttreatment change associated with modest enlargement that radiologically was not consistent with progressive disease (Table 2). A Kaplan-Meier curve depicting the observed PFS is shown in Fig. 1. None of the treated lesions underwent surgical resection as salvage treatment after radiation therapy.

**Table 2:** Treatment outcomes by course

Outcome	Count		
Response	Prior surgery (n=17)	No surgery (n=19)	Overall (n=36)
Symptom burden after radiation, n (%)			
Improved	9 (53%)	12 (63%)	21 (58%)
No change	7 (41%)	5 (26%)	12 (33%)
Worsening	1 (6%)	2 (11%)	3 (9%)
Radiographic response at last follow-up, n (%)			
Smaller	8 (47%)	10 (53%)	18 (50%)
No change	7 (41%)	8 (42%)	15 (42%)
Enlarged (including treatment effect)	1 (6%)	1 (5%)	2 (5%)
Not available	1 (6%)	0 (0%)	1 (3%)

Toxicities in both groups were found to have no association between prior surgery or lesion location. In total, 56% of the treated lesions demonstrated no acute or late toxicities. The most common acute toxicities were fatigue (17%), pain flare (17%), and dysphagia/dysgeusia (11%) shown in Table 3. The median thecal sac maximum (0.035 cm^3^) dose was 26.2 Gy (range, 0.4-30.2). There were no National Cancer Institute Common Terminology Criteria for Adverse Events (version 5.0) grade 3 or greater acute or long-term toxicities. One patient treated to 25 Gy in 5 fractions received a D0.35cc and D1.2cc dose to the spinal cord of 27.6 Gy and 24.6 Gy and experienced grade 2 thoracic cord myelitis at 6 months posttreatment which was managed with a 3-month course of dexamethasone followed by taper and with resolution of their symptoms.

**Table 3:** Acute toxicity outcomes by treatment course

Toxicity	Count		
Symptom	Prior Surgery (n = 17)	No Surgery (n = 19)	Overall (n = 36)
None	11 (65%)	9 (47%)	20 (56%)
Fatigue	3 (18%)	3 (16%)	6 (17%)
Pain Flare	2 (12%)	5 (21%)	6 (17%)
Dysphagia/Dysgeusia	1 (6%)	3 (16%)	4 (11%)
Headache	1 (6%)	0 (0%)	1 (3%)

## Discussion

The adoption of highly conformal image guided radiation therapy has allowed for the development of high-dose stereotactic treatment of spinal tumors.^15^^,^^16^ Although commonly used for oliogometastatic or radioresistant tumors of the spine, stereotactic radiosurgery (SRS) for the treatment of benign tumors is more controversial owing to the scarcity of long-term data for both efficacy and toxicity. In addition, the mainstay of treatment of benign spine tumors is surgical intervention, and so patients pursuing radiosurgery typically are nonsurgical candidates or defer surgery.^4^ Prior reports have explored the safety, efficacy, and long-term outcomes of spine radiosurgery for benign tumors; however, those patients were largely asymptomatic. This report sought to enrich the evidence base for radiosurgery for specifically symptomatic lesions.

SRS has been a mainstay of treatment for benign intracranial tumors, with excellent rates of local control in the definitive setting. Prior series of SRS for intracranial meningiomas and vestibular schwannoma point to 10-year control rates of greater than 90% with minimal risk of toxicity.^17^, ^18^, ^19^, ^20^ Assuming the biology of BST is similar to that of benign intracranial tumors, one would expect similar rates of response.

Prior studies of SBRT for BSTs have shown similar rates of local control and efficacy as for benign intracranial tumors, though these reports are limited by short-term follow-up.^6^^,^^8^ In the study by Gerszten et al,^6^ 73 patients with BSTs, which included meningioma, neurofibroma, and schwannoma, evidenced a local control of 100% with a median follow-up of approximately 3 years. Given the slow growth of these tumors longer-term outcomes are needed to ensure late failures are not being underrepresented. These results are similar to the current series which did not demonstrate any incidents of local failure, through the median follow-up of 49 months, makes estimation of long-term control challenging.

A contemporary series of 120 patients treated with SRS for BSTs with long-term follow-up have shown the 3, 5, and 10-year local recurrence rates to be 2%, 5%, and 12%, respectively.^5^ Similarly, a group of 184 patients with median follow-up of 63 months demonstrated a 1-, 5-, and 10-year local recurrence rate of 3%, 8%, and 10%, respectively.^21^ Additionally, both of these series demonstrate a low rate of toxicity including myelitis, which was found in 1% of patients in each series. In the current study, we noted a single episode of grade 2 myelitis managed with steroids, though given to the small cohort size this risk may be overrepresented. Other toxicities, including pain flare and fatigue, were similar among the series and occurred in approximately 10% to 15% of patients.

The focus of the current series is to evaluate the response of symptomatic BSTs, to further understand the role of radiation therapy as a means of symptom improvement. We demonstrated a 58% improvement in symptoms, and 33% stability of symptoms after radiation therapy. This is similar to prior series in which a subset of patients were symptomatic, and it was noted that 85% and 89% had improved or stable symptoms as seen in Table 4.^5^^,^^21^ These data provide further validation for SBRT in the setting of symptomatic tumors, and in patients who are not surgical candidates provides support for this treatment option.

**Table 4:** Outcome for symptom response

Study	Total lesions	Symptomatic lesions	Median follow up (mo)	Improved symptoms	Stable symptoms	5 year Local Control
Chin et al.	149	82*	49	26 (32%)	33 (40%)	95%
Taori et al.	207	156*	63	66 (42%)	67 (43%)	92%
Current Series	36	36	49	21 (58%)	12 (33%)	97%

⁎ Pain was only symptom considered

A limitation of this study is heterogeneous follow-up and symptom assessment of patients over the long term to assess changes in symptom burden and improvement over time. Notwithstanding, the effects of symptom improvement and stability are likely to be well captured within the first 12 months after treatment and may decline at a similar rate as local control. The systematic evaluation of symptoms burden before and after treatment may aid in further elucidating patients who have the highest likelihood of symptomatic improvement with radiation therapy.

## Conclusions

This study demonstrates in a cohort of patients who were not candidates for or refused surgery, SBRT can be used for the treatment of symptomatic BSTs and can achieve good symptomatic and radiographic control with acceptable toxicity in the upfront or salvage setting. Further characterization of these patients with validated prospective symptom tools would allow for further understanding of which patients may derive the greatest benefit, in addition to evaluating dose escalation as a means of improving response and tumor control.

## Declaration of interests

The authors declare that they have no known competing financial interests or personal relationships that could have appeared to influence the work reported in this paper.
